# The tryptophan catabolite or kynurenine pathway in major depressive and bipolar disorder: A systematic review and meta-analysis

**DOI:** 10.1016/j.bbih.2022.100537

**Published:** 2022-10-21

**Authors:** Abbas F. Almulla, Yanin Thipakorn, Asara Vasupanrajit, Ali Abbas Abo Algon, Chavit Tunvirachaisakul, Ashwan Abdulzahra Hashim Aljanabi, Gregory Oxenkrug, Hussein K. Al-Hakeim, Michael Maes

**Affiliations:** a Department of Psychiatry, Faculty of Medicine, Chulalongkorn University, Bangkok, Thailand; b Medical Laboratory Technology Department, College of Medical Technology, The Islamic University, Najaf, Iraq; c Iraqi Education Ministry, Najaf, Iraq; d Cognitive Impairment and Dementia Research Unit, Faculty of Medicine, Chulalongkorn University, Bangkok, Thailand; e Department of Psychiatry, Faculty of Medicine, University of Kufa, Iraq; f Department of Psychiatry Al-Hakeem General Hospital, Iraq; g Department of Psychiatry, Tufts University School of Medicine and Tufts Medical Center, Boston, MA, 02111, USA; h Department of Chemistry, College of Science, University of Kufa, Kufa, Iraq; i Department of Psychiatry, Medical University of Plovdiv, Plovdiv, Bulgaria; j Department of Psychiatry, IMPACT Strategic Research Centre, Deakin University, Geelong, Victoria, Australia

**Keywords:** Neuro-immune, Affective disorders, Inflammation, Oxidative and nitrosative stress, Psychiatry, Neurotoxicity

## Abstract

**Background:**

There is now evidence that affective disorders including major depressive disorder (MDD) and bipolar disorder (BD) are mediated by immune-inflammatory and nitro-oxidative pathways. Activation of these pathways may be associated with activation of the tryptophan catabolite (TRYCAT) pathway by inducing indoleamine 2,3-dioxygenase (IDO, the rate-limiting enzyme) leading to depletion of tryptophan (TRP) and increases in tryptophan catabolites (TRYCATs).

**Aims:**

To systematically review and meta-analyze central and peripheral (free and total) TRP levels, its competing amino-acids (CAAs) and TRYCATs in MDD and BD.

**Methods:**

This review searched PubMed, Google Scholar and SciFinder and included 121 full-text articles and 15470 individuals, including 8024 MDD/BD patients and 7446 healthy controls.

**Results:**

TRP levels (either free and total) and the TRP/CAAs ratio were significantly decreased (p < 0.0001) in MDD/BD as compared with controls with a moderate effect size (standardized mean difference for TRP: SMD = −0.513, 95% confidence interval, CI: −0.611; −0.414; and TRP/CAAs: SMD = −0.558, CI: −0.758; −0.358). Kynurenine (KYN) levels were significantly decreased in patients as compared with controls with a small effect size (p < 0.0001, SMD = −0.213, 95%CI: −0.295; −0.131). These differences were significant in plasma (p < 0.0001, SMD = −0.304, 95%CI: −0.415, −0.194) but not in serum (p = 0.054) or the central nervous system (CNS, p = 0.771). The KYN/TRP ratio, frequently used as an index of IDO activity, and neurotoxicity indices based on downstream TRYCATs were unaltered or even lowered in MDD/BD.

**Conclusions:**

Our findings suggest that MDD and BD are accompanied by TRP depletion without IDO and TRYCAT pathway activation. Lowered TRP availability is probably the consequence of lowered serum albumin during the inflammatory response in affective disorders.

## Highlights

• Major depressive disorder (MDD) and bipolar disorder (BD) are accompanied by central and peripheral tryptophan (TRP) depletion.
• The TRP catabolite (TRYCAT) pathway is not altered in mood disorders.
• There were no signs of increased TRYCAT-associated neurotoxicity with immunotoxic and oxidative properties.

## 1 Introduction

There is now robust evidence that activation of the immune-inflammatory response system (IRS) and the compensatory immune-regulatory system (CIRS) play an essential role in the pathophysiology of major depressive (MDD) and bipolar (BD) disorder (Almulla and Maes, 2022; Maes and Carvalho, 2018). Both disorders are characterized by elevated production of macrophage M1 and T helper (Th)1 cytokines including interleukin (IL)-1β, IL-6, IL-8, interferon (IFN)-γ, tumor necrosis factor (TNF)-α, whereas CIRS activation is indicated by elevated levels of anti-inflammatory products including Th-2 and Tregulatory (Treg) cytokines including IL-4 and IL-10 (Maes and Carvalho, 2018; Solmi et al., 2021). Both disorders are also accompanied by an acute-phase (AP) response with increased levels of positive AP proteins (APPs) such as haptoglobin and lowered levels of negative APPs such as albumin (Maes, 1993). Moreover, the activated IRS pathways in affective disorders are associated with activation of nitro-oxidative pathways with increased reactive oxygen and nitrogen species (RONS), and consequent lipid peroxidation and protein oxidation (Maes, 2022; Maes et al., 2011a). Activation of IRS and nitro-oxidative pathways are associated with the key features of affective disorders, including severity of illness, staging (reoccurrence of episodes) and suicidal behaviors including suicidal ideation and attempts (Maes, 2022; Maes et al., 2022b; Vasupanrajit et al., 2021). The current theory is that the neurotoxic effects of M1 and Th-1 cytokines and RONS cause neuro-affective toxicity with dysfunctions in brain connectome pathways that lead to staging and the phenome of MDD/BD (Maes, 2022; Maes et al., 2022b).

**Fig. 1:**
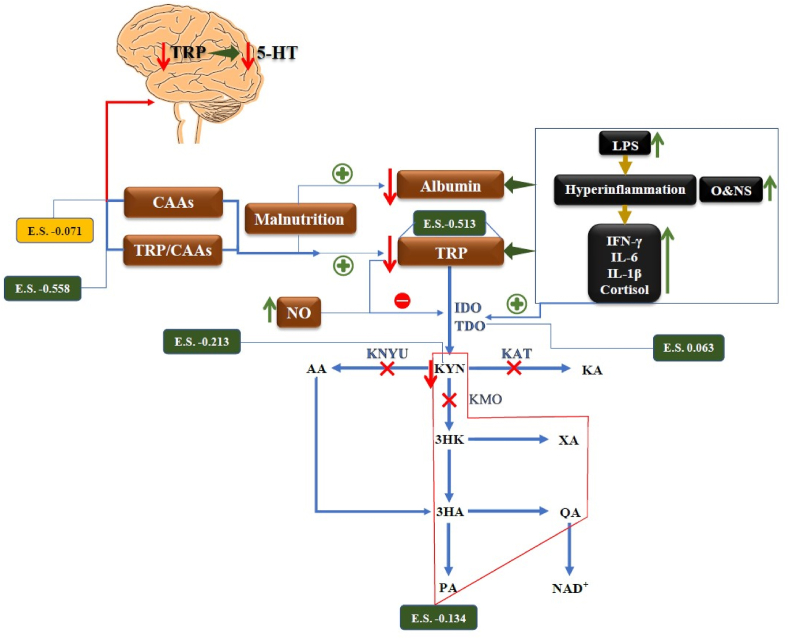
A Summary of tryptophan catabolite (TRYCAT) pathway in affective disorders. E.S.: Effect size, TRYCAT: Tryptophan catabolite, IFN-γ: Interferon-Gamma, IL-6: Interleukin 6, IL-1β: Interleukin-1 beta, O&NS: Oxidative and nitrosative stress, NO: Nitric Oxide, 5-HT: 5-Hydroxytryptamine, LPS: Lipopolysaccharides, CNS: Central nervous system, IDO: Indoleamine 2,3 dioxygenase, TDO: Tryptophan 2,3-dioxygenase, KAT: Kynurenine Aminotransferase, KMO: Kynurenine 3-monooxygenase, KYNU: Kynureninase, TRP: Tryptophan, KYN: Kynurenine, KA: Kynurenic Acid, 3HK: 3-Hydroxykynurenine, AA: Anthranilic Acid, XA: Xanthurenic Acid, 3HA: 3-Hydroxyanthranilic Acid, PA: Picolinic Acid, QA: Quinolinic Acid.

Activation of IRS and nitro-oxidative pathways has a number of major detrimental consequences including depletion of tryptophan (TRP) in peripheral blood and increases in levels of neurotoxic tryptophan catabolites (TRYCATs) (Maes, 2015; Maes et al., 2011d). Since tryptophan binds tightly to albumin, the decreased levels of albumin during the acute phase or IRS response in affective disorders may result in a decrease in total TRP levels in peripheral blood (Maes et al., 2011d). Moreover, products of the IRS and nitro-oxidative stress response during MDD/BD may activate indoleamine-2,3-dioxygenase (IDO), the rate limiting enzyme of the TRP catabolite (TRYCAT) pathway, which may cause increased TRYCATs production and lower TRP thereby diverting TRP from serotonin synthesis (Maes et al., 2011d) (Fig. 1). Reactive oxygen species (ROS), IL-1β, TNF-α, IFN-α, IFN-γ (Maes et al., 2011d) and lipopolysaccharides (LPS), generated by translocation of Gram-negative bacteria (Maes et al., 2008), may all stimulate IDO. While IDO is active in immune cells (macrophages and dendritic cells) and brain cells (e.g. astrocytes), tryptophan-2,3-dioxygenase (TDO) is activated by glucocorticoids and is expressed primary in the liver where it converts TRP to the same TRYCATs (Maes et al., 1991a, 2011d).

Overall, stimulation of the TRYCAT pathway has CIRS functions including anti-inflammatory and antioxidant effects in part through reductions in TRP, which results in negative immune-regulatory, antiproliferative and antimicrobial effects, and elevations in TRYCATs which have anti-inflammatory and antioxidant effects (Almulla and Maes, 2022; Maes et al., 2011d). For example, kynurenic acid (KA), xanthurenic acid (XA) and quinolinic acid (QA) may exert anti-inflammatory effects through their ability to decrease the IFN-γ/IL-10 ratio (Almulla and Maes, 2022; Maes, 2015; Maes et al., 2007). On the other hand, increases in downstream TRYCATs may cause neurotoxicity: kynurenine (KYN), QA, picolinic acid (PA) and XA have neurotoxic effects, and 3-hydroxyanthranilinc acid (3HA), 3-hydroxykynurenine (3HK) and QA may induce oxidative stress (Reyes Ocampo et al., 2014; Santamaría et al., 2001; Smith et al., 2009).

Previous research (Maes et al., 1990, 1995; Marx et al., 2021) and meta-analysis (Ogawa et al., 2014) revealed that patients with MDD and BD show reduced TRP levels. In mood disorders, low TRP has been reported to be a biomarker of IRS activation and the acute phase response (Maes, 2015), whilst some but not all reports show increased TRYCATs levels in MDD/BD. Ogyu et al. showed that drug-free depressed patients have diminished levels of KYN and KA and increased QA levels (Ogyu et al., 2018). Furthermore, Arnone et al. reported that MDD patients demonstrated lowered KYN level compared with healthy controls (Arnone et al., 2018). Marx et al. found that MDD was accompanied by decreased TRP, KYN and KA levels and that BD patients showed reduced TRP and KA levels (Marx et al., 2021). Hebbrecht et al. reported lowered peripheral TRP, KYN, KA levels in BD patients compared to healthy controls (Hebbrecht et al., 2021).

TRYCATs with neurotoxic activities including KYN and 3-HK are suggested to play a key role in IFN-α induced major depression (Bonaccorso et al., 2001; Maes et al., 2001a). Indeed, the onset of these IFN-α-induced depressive symptoms is more strongly associated with the production of KYN (a neurotoxic TRYCAT) and an increased ratio of KYN to KA, a neuroprotective TRYCAT, than with lowered TRP levels (Bonaccorso et al., 2001, 2002; Maes, 2015; Maes et al., 2001b; Wichers et al., 2005). By inference, it was thought that also in MDD/BD, which is characterized by lowered plasma/serum TRP, increased neurotoxicity due to IDO stimulation could be the major culprit (Bonaccorso et al., 2002).

Nevertheless, the abovementioned meta-analyses did not include the levels of free or total TRP and their competing amino acids (CAAs), namely valine, tyrosine, leucine, isoleucine and phenylalanine, in order to evaluate the TRP/CAAs ratio which is a more adequate index reflecting TRP availability to the brain than plasma/serum TRP (Lucini et al., 1996). In addition, these meta-analyses did not provide sufficient evidence to determine whether affective disorders are related with alterations in downstream neurotoxic TRYCATs due to activated IDO activity. Finally, we recently discovered that the serum and plasma TRYCAT results in schizophrenia are dissociated from central nervous system (CNS) findings, and that there are significant discrepancies in the correlations between schizophrenia and KYN in serum versus plasma (Almulla et al., 2022c).

Hence, we conducted a systematic review and meta-analysis to comprehensively examine central and peripheral (free and total) TRP, the sum of CAAs and the TRP/CAAs ratio (index for the availability of TRP to the brain) along with indices of IDO, Kynurenine aminotransferase (KAT) and Kynurenine 3-monooxygenase (KMO) enzyme activities, and the levels of downstream neurotoxic TRYCATs in both MDD and BD in CNS, serum and plasma.

## 2 Materials and methods

We conducted the current meta-analysis to examine the peripheral (serum and plasma) and central (cerebrospinal fluid,CSF and brain) levels of TRP, KYN, KA and 3HK as well as some ratios namely KYN/TRP which reflects the IDO enzyme activity, KA/KYN (KAT enzyme activity and 3HK/KYN (KMO enzyme activity) along with the neurotoxic TRYCAT composite (KYN+3HK+3HA + QA + XA + PA) in patients with MDD and BD. In the same patients we also assessed peripheral level of CAAs and TRP/CAAs ratio.

The methodology of this study was based on the guidelines of a) Preferred Reporting Items for Systematic Reviews and Meta-Analyses (PRISMA) 2020 (Page et al., 2021), b) Cochrane Handbook for Systematic Reviews and Interventions (Higgins JPT et al., 2019), c) the Meta-Analyses of Observational Studies in Epidemiology (MOOSE).

### 2.1 Search strategy

The 10th of January 2022 marked the beginning of our examination of the electronic databases PubMed/MEDLINE, Google Scholar, and SciFinder. To collect all the publications pertaining to TRP and TRYCATs in affective disorders, we used the keywords and mesh terms provided in Table 1 of the Electronic supplementary file (ESF). The database search concluded at the end of March 2022. Nevertheless, we verified the reference lists of all eligible papers and previous meta-analyses to prevent the omission of pertinent publications.

**Table 1:** The outcomes and number of patients with affective disorders and healthy control along with the side of standardized mean difference (SMD) and the 95% confidence intervals with respect to zero SMD.

Outcome profiles	n studies	Side of 95% confidence intervals				Patient Cases	Control Cases	Total number of participants
		<0	Overlap 0 and SMD <0	Overlap 0 and SMD >0	>0			
TRP	106	48	48	8	2	5753	5288	11041
TRP/CAAs	18	7	11	0	0	423	607	1030
CAAs	14	1	6	4	3	330	503	833
KYN	67	19	30	15	3	4813	4356	9169
KYN/TRP	68	5	15	42	6	6007	5437	11444
(KYN+3HK+3HA + XA + QA + PA)	82	22	35	18	7	5392	4853	10245
KA/KYN	70	5	36	21	8	4987	4322	9309
KA	55	16	25	10	4	3905	3464	7369
3HK	26	4	10	11	1	1364	1244	2608

TRP: Tryptophan, KYN: Kynurenine, KA: Kynurenic acid, 3HK: 3-Hydroxykynurenine, 3HA: 3-Hydroxyanthranilic acid, XA: Xanthurenic acid, QA: Quinolinic acid, PA: Picolinic acid, AA: Anthranilic acid, SMD: Standardized Mean Difference, CAAs: Competing amino acids (Valine + Phenylalanine + Tyrosine + Leucine + Isoleucine).

### 2.2 Eligibility criteria

Publication in peer-reviewed journals and English language served as the key inclusion criteria for the papers in our meta-analysis. However, we also searched for grey literature, papers written in Thai, French, Spanish, German, Italian, and Arabic, although all included articles were in English and peer-reviewed papers. In addition, we set inclusion criteria for observational case-control and cohort studies that evaluated the levels of TRP and TRYCATs peripherally in serum and plasma and centrally namely in CSF and brain tissues (post-mortem studies). Patients should be diagnosed in accordance with the Diagnostic and Statistical Manual of Mental Disorders (DSM) or International Classification of Diseases (ICD) criteria. Thirdly, we considered longitudinal studies that gave the baseline values of the relevant biomarkers. We excluded a) animal, genetics, and translational studies, as well as systematic reviews and meta-analyses; b) studies lacking a control group; c) studies reporting on saliva, hair, whole blood, and platelet-rich plasma samples; d) duplicate studies; and e) articles lacking the mean and standard deviation (SD) or standard error (SE) values of the measured biomarkers. Nonetheless, we asked that authors furnish us with mean (SD) values when they did not provide mean with SD or SEM values. Without more information from the authors, we estimated the mean (SD) values from the median values using the Wan et al. (2014) technique or computed the mean (SD) values from graphical formats using the Web Plot Digitizer (https://automeris.io/WebPlotDigitizer/).

### 2.3 Primary and secondary outcomes

In the primary outcome of the current meta-analysis, we investigated the TRP and KYN levels along with IDO enzyme activity by examining the KYN/TRP ratio in affective disorders patients versus healthy control, in addition to CAAs and TRP/CAAs ratio (see Table 1). Secondary outcomes involved determining the KA/KYN and 3HK/KYN ratios as indices of KAT and KMO enzymes activity respectively besides the neurotoxic TRYCAT composite (KYN+3HK+3HA + QA + XA + PA) and the solitary levels of other TRYCATs namely KA and 3HK.

### 2.4 Screening and data extraction

The first two authors (AA and YT), conducted a basic evaluation of the relevant studies according to the stated inclusion criteria by examining the titles and abstracts to determine the eligibility of each research for inclusion in our meta-analysis. Then, we downloaded the complete texts of papers that met our inclusion criteria, excluding research that did meet our exclusion criteria. They used a predefined Excel file with the mean, standard deviation, and other relevant information of the included research. The final spreadsheet was double-checked by YT and AA, who contacted the last author (MM) in case of any discrepancies.

The predefined Excel file comprised of the authors' names, the publication dates of the studies, the names and mean and standard deviation (SD) values of evaluated TRP and TRYCATs, and the sample sizes of both patient and healthy control groups. In addition, the research design, sample type (serum, plasma, CSF, and brain tissues), psychiatric assessment scales, and participants' demographic data, including mean (SD) age, gender, and study location, were included. Furthermore, the quality of the methodology was evaluated using the immunological confounder scale (ICS) (Andrés-Rodríguez et al., 2020). The last author modified the ICS to make it consistent with the TRYCATs research. Quality paper controls consists of two scoring scales, namely the quality and redpoints scales, which are detailed in ESF, Table 2. These rating scales were largely used (Almulla et al., 2022a, 2022b, 2022c) to evaluate the methodological quality of the publications that assessed the levels of TRYCATs in individuals with affective disorders. The score on the quality scale varied from 0 to 10, representing lower to better quality, and it focused largely on sample size, control of confounders, and sampling duration. While the redpoints scale was primarily intended to anticipate probable bias in the outcomes of TRYCATs and research designs by examining the amount of control over the important confounders. The maximum degree of control was attained when the overall score was zero; conversely, a score of twenty-six shows that the confounding variables were not considered.

**Table 2:** Results of meta-analysis conducted on several outcome (TRYCATs) variables with combined different media and separately.

Outcome feature sets	n	Groups	SMD	95% CI	z	p	Q	df	p	I^2^ (%)	τ^2^	Τ
TRP*	106	Overall	−0.513	−0.611; −0.414	−10.215	<0.0001	553.03	105	<0.0001	81.01	0.188	0.434
TRP/CAAs*	18	Overall	−0.558	−0.758; −0.358	−5.464	<0.0001	31.58	17	0.017	46.18	0.077	0.277
CAAs*	14	Overall	−0.071	−0.332; 0.189	−0.538	0.591	33.66	13	0.001	61.37	0.133	0.365
KYN	67	Overall	−0.213	−0.295; −0.131	−5.103	<0.0001	222.73	66	<0.0001	70.36	0.028	0.279
	5	CNS	0.178	−0.274; 0.630	0.771	0.441	16.49	4	0.002	75.74	0.191	0.437
	37	Plasma	−0.304	−0.415; −0.194	−5.392	<0.0001	103.19	36	<0.0001	65.11	0.065	0.255
	25	Serum	−0.125	−0.251; 0.002	−1.927	0.054	69.82	24	<0.0001	65.62	0.060	0.245
KYN/TRP*	68	Overall	0.063	−0.029; 0.154	1.332	0.183	310.963	67	<0.0001	78.45	0.100	0.316
(KYN+3HK+3HA + XA + QA + PA)*	82	Overall	−0.134	−0.232; −0.036	−2.672	0.008	401.60	81	<0.0001	79.83	0.145	0.380
KA/KYN*	70	Overall	0.024	−0.060; 0.107	0.559	0.576	215.84	69	<0.0001	68.03	0.073	0.270
KA	55	Overall	−0.234	−0.344; −0.124	−4.157	<0.0001	260.27	54	<0.0001	79.25	0.132	0.363
	9	CNS	0.140	−0.135; 0.415	0.998	0..318	26.05	8	<0.0001	69.29	0.114	0.338
	23	Plasma	−0.379	−0.552; −0.206	−4.288	<0.0001	121.61	22	<0.0001	81.91	0.126	0.355
	23	Serum	−0.237	−0.404; −0.070	−2.775	0.006	78.52	22	<0.0001	71.98	0.114	0.338
3HK*	26	Overall	−0.030	−0.187; 0.127	−0.379	0.705	88.12	25	<0.0001	71.63	0.114	0.337

*: No significant differences between central nervous system (CNS, cerebrospinal fluid + brain tissues), serum and plasma.

TRP: Tryptophan, KYN: Kynurenine, KA: Kynurenic acid, 3HK: 3-Hydroxykynurenine, 3HA: 3-Hydroxyanthranilic acid, XA: Xanthurenic acid, QA: Quinolinic acid, PA: Picolinic acid, AA: Anthranilic acid, SMD: Standardized Mean Difference, CI: Confidence intervals. TRYCATs: Tryptophan catabolites, CAAs: Competing amino acids (Valine + Phenylalanine + Tyrosine + Leucine + Isoleucine).

### 2.5 Data analysis

The present meta-analysis was conducted using the CMA V3 program and the PRISMA criteria (ESF, Table 3). To perform a meta-analysis on TRP or a particular TRYCAT, at least three studies on that TRYCAT were necessary. The neurotoxicity index and ratios were compared between patients and controls by computing the mean values of the outcomes while assuming dependence. Thus, we compared the neurotoxic TRYCATs profile of patients with affective disorders and healthy controls by entering the relevant TRYCATs in the meta-analysis, and we analyzed the KYN/TRP, KA/KYN and 3HK/KYN ratios as an indexes for IDO, KAT and KMO enzyme activities respectively (Almulla et al., 2022c). IDO enzyme activity is estimated by selecting a positive effect size direction for increasing KYN and a negative direction for lowered TRP levels in patients, KAT activity was assessed by setting KA as positive and KYN as negative, KMO by setting 3HK as positive and KYN as negative, and the TRP/CAAs ratio by setting TRP as positive and CAA as negative. We utilized the random-effects model with constrained maximum likelihood to pool the effect sizes since participants characteristics were not homogeneous across trials in the current meta-analysis. We report the effect size as the standardized mean difference (SMD) with 95 percent confidence intervals (95% CI), and statistical significance was defined as a two-tailed p-value less than 0.05. The effect size was described as large, moderate, and small based on the SMD values of 0.80, 0.50, and 0.20, respectively (Cohen, 1988). In accordance with prior meta-analyses (Almulla et al., 2022c; Vasupanrajit et al., 2022), heterogeneity was determined by calculating tau-squared statistics, although we also show the Q and I^2^ metrics. To identify the causes of heterogeneity in the present meta-analysis, a meta-regression was conducted. We used subgroup analysis to identify differences in TRP and TRYCATs in different media, including in CNS (brain tissues + CSF), serum and plasma, as well as the distinctions between MDD and BD. Each of the above categories serves as a unit of analysis. Since there are no indications of statistically significant differences between studies that measured CSF and brain tissues in the biomarkers, we merged the CSF and brain tissues under the umbrella name CNS. We show the results in the combined study group of affective disorders (MDD + BD), and when there are significant differences, we show the results in MDD and BD, separately. We conducted sensitivity analyses using the leave-one-out approach to examine the robustness of the impact sizes and heterogeneity across studies. Publication bias was investigated using the fail-safe N technique, continuity-corrected Kendall tau, and Egger's regression intercept, using one-tailed p-values for the last two approaches. In the face of an asymmetry shown by Egger's test, we used the trim-and-fill approach developed by Duval and Tweedie to impute the missing studies and compute adjusted effect sizes (Duval and Tweedie, 2000a, 2000b). To discover small study effects, we also used funnel plots (study precision vs SMD), which concurrently show observed and imputed missing values.

**Table 3:** Results on publication bias.

Outcome feature sets	Fail safe n	Z Kendall's τ	p	Egger's *t*-test (df)	p	Missing studies (side)	After Adjusting
TRP (Overall)	−20.06	2.42	0.007	5.62(104)	<0.0001	22(Right)	−0.324(-0.427;-0.222)
TRP/CAAs (Overall)	−7.56	1.89	0.029	2.66(16)	0.008	6 (Right)	−0.344(-0.568; −0.120)
CAAs (Overall)	−0.992	0.43	0.330	0.407(12)	0.345	1(Right)	−0.008(-0.283; 0.265)
KYN (Overall)	−8.03	0.270	0.393	0.425(65)	0.336	11(Right)	−0.108(-0.199;-0.016)
KYN (CNS)	1.99	<0.0001	0.500	1.10(3)	0.174	0	–
KYN (Plasma)	−8.82	0.013	0.494	0.892(35)	0.188	0	–
KYN (Serum)	−2.18	0.925	0.177	0.659(24)	0.257	3(Right)	0.067(-0.195; 0.061)
KYN/TRP (Overall)	1.35	0.820	0.205	4.02(66)	0.00008	20(Left)	−0.074(-0.160; 0.010)
(KYN+3HK+3HA + XA + QA + PA) (Overall)	−5.75	0.176	0.430	0.007(80)	0.497	21(Right)	0.056(-0.048; 0.161)
KA/KYN (Overall)	0.965	0.192	0.423	0.219(68)	0.413	18(Right)	0.149(0.065; 0.233)
KA (Overall)	−8.66	0.392	0.347	0.304(53)	0.381	14(Right)	−0.074(-0.195; 0.047)
KA (CNS)	1.94	0.312	0.377	0.433(7)	0.338	2(Right)	0.280(-0.001; 0.563)
KA (Plasma)	−9.08	1.10	0.133	1.47(21)	0.077	0	–
KA (Serum)	−5.52	0.422	0.336	1.43(21)	0.082	6(Right)	−0.123(-0.284; 0.037)
3HK (Overall)	−0.887	0.837	0.201	0.958(24)	0.173	1(Right)	0.010(-0.150; 0.181)

TRP: Tryptophan, KYN: Kynurenine, KA: Kynurenic acid, 3HK: 3-Hydroxykynurenine, 3HA: 3-Hydroxyanthranilic acid, XA: Xanthurenic acid, QA: Quinolinic acid, PA: Picolinic acid, AA: Anthranilic acid, CAAs: Competing amino acids (Valine + Phenylalanine + Tyrosine + Leucine + Isoleucine).

## 3 Results

### 3.1 Search results

**Fig. 2:**
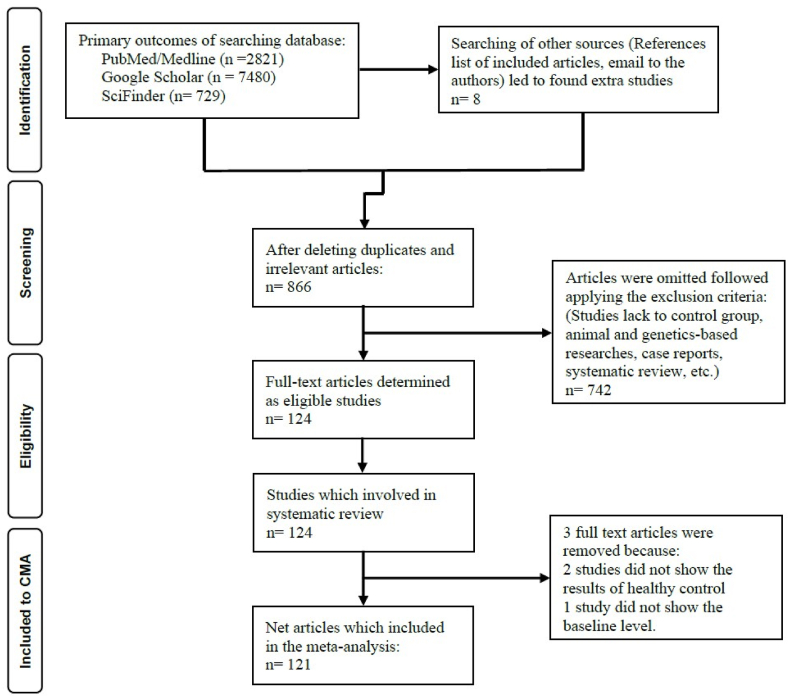
The PRISMA flow chart.

Fig. 2 shows the PRISMA flow chart with the overall search outcomes and the number of included and omitted articles. We investigated 11038 articles of the initial searching processes, which relied on our specific keywords and mesh terms (as listed in ESF, Table 1). However, we refined the search's results and eliminated 10172 duplicate and irrelevant studies. After applying our inclusion-exclusion criteria, 124 full-text eligible articles were included in the current systematic review. Due to exclusion criteria mentioned in ESF, table 4, three out of these 124 studies were excluded. Hence, the current meta-analysis involved 121 studies (Aarsland et al., 2019; Achtyes et al., 2020; Anderson et al., 1990; Baranyi et al., 2017; Bay-Richter et al., 2015; Birner et al., 2017; Bradley et al., 2015; Brundin et al., 2016; Busse et al., 2015; Carrillo-Mora et al., 2020; Castillo et al., 2019; Cathomas et al., 2021; Chiaroni et al., 1990; Chiu et al., 2021; Cho et al., 2017; Clark et al., 2016; Colle et al., 2020; Coppen et al., 1972; Coppen et al., 1973; Cowen et al., 1989; Czermak et al., 2008; Dahl et al., 2015; DeMyer et al., 1981; DeWitt et al., 2018; Doolin et al., 2018; Ebesunun et al., 2012; Erabi et al., 2020; Erhardt et al., 2013; Gabbay et al., 2010; Georgin-Lavialle et al., 2016; Gerner et al., 1984; Guicheney et al., 1988; Hayward et al., 2005; Healy et al., 1982; Heilman et al., 2019; Hennings et al., 2013; Hoekstra et al., 2006; Hoekstra et al., 2001; Hu et al., 2017; Hüfner et al., 2015; Hughes et al., 2012; Joseph et al., 1984; Karege et al., 1994; Krause et al., 2019; Krause et al., 2017; Kuwano et al., 2018; Liu et al., 2018; Lucca et al., 1992; Maes et al., 1995; Maes et al., 2011b; Maes et al., 1990; Maes et al., 1993; Maes and Rief, 2012; Maes et al., 1997b; Maes et al., 1996; Manjarrez-Gutierrez et al., 2009; Mauri et al., 2001; Mauri et al., 1998; Meier et al., 2016; Menna-Perper et al., 1983; Milaneschi et al., 2021; Miller et al., 2006; Moaddel et al., 2018; Møller, 1993; Moller et al., 1976; Møller et al., 1982; Moreno et al., 1999; Moreno et al., 2013; Mukherjee et al., 2018; Murata et al., 2020; Myint et al., 2007a; Myint et al., 2007b; Nikkheslat et al., 2015; Ogawa et al., 2018; Olsson et al., 2010; Ortiz et al., 1993; Öztürk et al., 2021; Pan et al., 2018; Paul et al., 2022; Pinto et al., 2012; Platzer et al., 2017; Poletti et al., 2019; Poletti et al., 2018; Pompili et al., 2019; Porter et al., 2003; Price et al., 1991; Quak et al., 2014; Quintana, 1992; Reininghaus et al., 2014; Rief et al., 2004; Roiser et al., 2009; Russ et al., 1990; Ryan et al., 2020; Sakurai et al., 2020; Savitz et al., 2015a; Savitz et al., 2015b; Savitz et al., 2015c; Savitz et al., 2015d; Schwieler et al., 2016; Sellgren et al., 2019; Shaw et al., 1980; Song et al., 1998; Sorgdrager et al., 2017; Steen et al., 2020; Steiner et al., 2011; Sublette et al., 2011; Sun et al., 2020; Teraishi et al., 2015; Trepci et al., 2021; Umehara et al., 2017; van den Ameele et al., 2020; Veen et al., 2016; Wood et al., 1978; Wu et al., 2018a; Wu et al., 2018b; Wurfel et al., 2017; Xu et al., 2012; Yoshimi et al., 2016; Young et al., 2016; Zhou et al., 2018; Zhou et al., 2019). Ten of the eligible articles examined TRP and TRYCATs in MDD and BD within the same study. Three studies assessed central and peripheral levels of TRP and TRYCTAs. Furthermore, one study involved two separate cohorts of patients and healthy controls. Hence, in the present meta-analysis, the overall effect size was pooled from 135 (16 CNS, 84 plasma, 35 serum) studies, namely 110 studies (10 CNS, 71 plasma, 29 serum) in MDD patients and 25 studies (6 CNS, 13 plasma, 6 serum) in BD patients. The total recruited number of individuals was 15470 in the present meta-analysis, distributed as 8024 patients with affective disorders and 7446 control subjects. The age of the participants extended from 16 to 69 years.

As shown in ESF, Table 5, our systematic review found that high-performance liquid chromatography (HPLC) was the most often used method for measuring TRP and TRYCATs in CNS, serum, and plasma, as it has been deployed in 50 papers. Numerous nations contributed to the included studies; the United States contributed the most with 29 studies, followed by the United Kingdom with 10 studies; the remainder of the contributors were as follows: 9 from Sweden, 8 are from China and Germany, 7 are from Belgium, 6 are from Japan, the Netherlands, and Italy, 5 are from Austria, 4 are from Ireland, 3 are from Denmark, France, Mexico and Switzerland 2 are from Norway, South Korea and Spain, and only one is from Taiwan, Turkey, Brazil, Canada, and Nigeria. We analyzed the quality and redpoints whose median (min-max) ratings were 5 (min = 1, max = 8,75), 13,5 (min = 4,5, max = 23), respectively, and the results are presented in Table 5 of ESF.

### 3.2 Primary outcome variables

#### 3.2.1 TRP and the TRP/CAAs ratio

The present meta-analysis identified TRP measurements in 106 research papers. Table 1 shows that in 48 studies the CI were entirely negative of zero, whereas there were only 2 studies which showed CI intervals that were entirely positive of zero. There were 56 studies that showed CI overlapping with zero and of those 48 showed SMD values smaller than zero, and 8 papers showed SMD values greater than zero. In individuals with affective disorders, TRP was significantly decreased with a moderate effect size of −0.513 as shown in Table 2. There was some publication bias as shown in Table 3 with 22 papers missing on the right side of the funnel plot. Nevertheless, after imputing the missing studies, the adjusted point estimate remained significant at SMD = −0.324.

We conducted a subgroup analysis to investigate the differences between total and free TRP levels in patients versus controls. The results indicated no significant difference (p = 0.176) between total and free TRP levels, whilst both were decreased in affective disorders patients.

Table 2 shows that the TRP/CAAs ratio was significantly lower in patients than in controls with a modest effect size of −0.558. Table 3 shows a bias with 6 studies missing on the right side of the funnel plot and imputing these studies lowered the SMD value although it remained significant. CAAs results were obtained from 14 studies. Table 1, Table 2 show that CAAs levels were not significantly different between patients and controls.

#### 3.2.2 KYN and the KYN/TRP ratio

We included 67 (5 CNS, 37 plasma and 29 serum) KYN studies in the present systematic review. Table 1 shows the CI distributions of the KYN and KYN/TRP data. Table 2 shows that KYN was significantly lower in patients than in controls with a small effect size. Nevertheless, group analysis (using CNS, serum and plasma as unit of analysis) showed a significant difference (p = 0.025) between CNS, plasma, and serum and that KYN levels were significantly reduced in plasma albeit with a modest impact size, but not in the CNS or serum. Egger's regression and Kendall's tau revealed publishing bias in serum and imputing 3 studies to the right reduced the SMD value to 0.067, whereas there was no bias in the CNS and plasma studies (see Table 3). No significant difference was found in the KYN/TRP ratio between patients and controls as shown in Table 2.

### 3.3 Secondary outcome variables

The effect size of the neurotoxicity composite (KYN+3HK+3HA + PA + XA + QA) was obtained from 82 studies. Table 2 shows that patients with affective disorders have a significantly reduced neurotoxicity composite index, albeit with a small effect size (SMD = −0.134). There was evidence of bias with 21 studies on the right and adjusting the effect size for these studies changed the results to non-significant (see Table 3). Table 2 shows no significant difference in the KA/KYN ratio between patients and controls, although after imputing 18 missing studies on the right site, the KA/KYN ratio was increased (see Table 3). There were significant differences in KA results between CNS, plasma and serum with plasma and serum KA being significantly decreased in patients. Nevertheless, after imputing missing studies the differences in serum KA were no longer significant. Table 2 shows no significant differences in 3HK between the groups.

### 3.4 Meta-regression analyses

We conducted meta-regression analysis to identify the most likely causes of the heterogeneity in TRP and TRYCAT data. As demonstrated in ESF, table 6, the unmedicated status affected TRP and CAAs. In addition, TRP was impacted by male gender and sample size. Age and severity are likely other factors that impact part of the variability. The higher number of studies compared to prior meta-analyses influenced all outcomes in ESF Table 6 except 3HK, CAAs, and TRP/CAAs ratio. There were no significant effects of quality and redpoints scores on the measured biomarkers. ESF, Table 6 shows that female sex impacted TRP levels in drug-naïve patients.

## 4 Discussion

### 4.1 Tryptophan availability to the brain

The first major finding of this large-scaled systematic review is that total and free TRP and the TRP/CAAs ratio were significantly lower with a moderate effect size in MDD and BD patients than in controls and that there were no differences between MDD and BD. The current findings are consistent with the results of prior meta-analyses (Marx et al., 2021; Ogawa et al., 2014), although the meta-analysis conducted by Arnone et al. found unaltered TRP in MDD and BD patients (Arnone et al., 2018). Ogawa et al. examined plasma TRP levels only (Ogawa et al., 2014), whilst we investigated CNS, plasma and serum levels. The amount of TRP which will reach the brain depends not only on the concentrations of peripheral, total and free TRP, but also on CAAs and the TRP/CAAs ratio (Pardridge, 1979; Yuwiler et al., 1977). Brain TRP concentrations are influenced by CAAs since the latter are competing with TRP for transport through the large amino acid transporter 1 (LAT 1) of the blood brain barrier (BBB) (Fernstrom et al., 1973; Pardridge, 1979). In this respect, our study found that the CAAs levels were unaltered in MDD/BD indicating that the lowered TRP/CAAs ratio is explained by lowered TRP. Such findings were indeed reported in previous studies (DeMyer et al., 1981; Maes et al., 1993, 1996; Song et al., 1998).

### 4.2 KYN and IDO

The second major finding of the current research is that peripheral KYN levels were significantly lower in patients than in controls, whereas the KYN/TRP ratio was unaltered. The present KYN findings are consistent with preceding meta-analyses (Bartoli et al., 2021; Hebbrecht et al., 2021; Marx et al., 2021; Ogyu et al., 2018), although Arnone et al. reported normal KYN levels in BD patients (Arnone et al., 2018). Nevertheless, previous studies were performed on a smaller number of studies and did not consider possible differences among central, serum and plasma KYN assays. In this respect, subgroup analysis revealed that peripheral KYN levels were lowered in the plasma of patients but not in their serum. All in all, since KYN is not altered in serum and CNS and since plasma KYN levels are more difficult to interpret (see below), the KYN data indicate decreased or unchanged levels in the major affective mood disorders.

### 4.3 TRYCAT neurotoxicity in affective disorders

A third major finding of the current systematic review is that patients with affective disorders showed no significant changes in the neurotoxicity index (after correcting for possible bias) comprising KYN, 3HK, 3HA, PA, XA and QA. Moreover, patients showed a significant increase in the KA/KYN ratio (an indicator of lowered KYN-associated neurotoxicity) and a reduction in KA in peripheral blood only. Previous meta-analysis also reported a decrease in peripheral KA levels, although they did not measure CNS KA levels (Bartoli et al., 2021; Hebbrecht et al., 2021; Marx et al., 2021; Ogyu et al., 2018). The current findings suggest that the IDO enzyme and production of neurotoxic TRYCATs are not upregulated in patients with mood disorders, which partially contradicts the IDO theory of affective disorders, postulating that IDO stimulation with decreased TRP and elevated neurotoxic TRYCATs is involved in the pathophysiology of MDD/BD (Maes et al., 2011d).

### 4.4 Heterogeneity

In the current meta-analysis, we found a high degree of heterogeneity in the TRP and TRYCATs data, and our meta-regression and group analyses uncovered significant sources of heterogeneity. Discrepancies between measurements in CNS, plasma and serum KYN were also detected in a previous meta-analysis in schizophrenia (Almulla et al., 2022c). In that paper, we have discussed that plasma is not the most accurate medium for determining KYN (and other TRYCATs) because plasma assays are more susceptible to pre-analytical and analytical errors (Almulla et al., 2022c) resulting from a) probable degradation of KYN and TRP in response to the presence of carbonyl-containing compounds, namely EDTA (Bellmaine et al., 2020), b) dilutional effects (particularly on small amounts of analytes) of anticoagulants in plasma tubes (Sotelo-Orozco et al., 2021), and c) increased α-amino products due to EDTA decomposition in high temperatures (Parvy et al., 1983). Moreover, previous studies recommended serum to assay TRP since the anticoagulants within plasma tubes may contaminate the measurements, particularly when using HPLC and spectrophotometers techniques (Davidson, 2002; Kulkarni et al., 2016). In addition to the impact of differences between serum, plasma, and CNS, other important sources of heterogeneity include a) the medicated status of the patients, with unmedicated patients exhibiting larger effect sizes in most biomarkers compared to treated patients, and b) sex which impacts the KYN/TRP ratio, and c) to a lesser extent, age and sample size (see ESF, Table 6).

### 4.5 Interpretation of the results

The inflammatory-IDO-neurotoxicity theory of affective disorders was developed based on mechanistic studies that IFN-α-based immunotherapy causes depression and that increased IRS responses and production of neurotoxic TRYCAT levels are directly related to the onset of this type of depression (Bonaccorso et al., 2002). Nonetheless, our negative results concerning TRYCAT and IDO levels do not support the theory that activation of IDO/TDO is involved in MDD/BD (Maes, 2015). While the TRYCAT pathway is activated during severe IRS responses, such as acute COVID-19 infection (Almulla et al., 2022b) and IFN-α therapy (Bonaccorso et al., 2002), no such changes may be observed in conditions of mild chronic inflammation, such as MDD/BD (this study) and Alzheimer's disease (Almulla et al., 2022a). Since plasma/serum TRP concentrations are around 50 μmol/L while KYN concentrations are around 3 μmol/L, TRP is abundantly available as a substrate for KYN formation. Therefore, it is plausible that IDO (TDO) may be inhibited in MDD/BD rather than that lowered substrate availability determines the downregulation of the pathway. First, since the substrate TRP is reduced in MDD/BD patients, the IDO enzyme may be self-regulating and transformed to an inactive state (Nelp Micah et al., 2018). When TRP concentrations are low, catalytically inactive ferric IDO1 may accumulate during turnover and the enzyme may autooxidize (Booth et al., 2015). Second, the IDO enzyme is inhibited by nitric oxide (NO), which is significantly increased in MDD and BD (Maes et al., 2019c; Savaş et al., 2002; Talarowska et al., 2012).

All in all, our results suggest that mood disorders are characterized by lowered TRP levels while IDO and the TRYCAT pathway are not stimulated. The majority of TRP in the circulation is bound to albumin (the total TRP pool) and, therefore, any changes in serum albumin will impact total TRP levels (Fernstrom et al., 1973; Mc and Oncley, 1958). Some studies found that serum albumin levels were lower in people with depression and that there was a link between serum albumin and total serum/plasma TRP (Liu et al., 2015; Maes et al., 1991b, 1996, 1997a). As such, reduced albumin levels, due to the acute phase or mild chronic IRS response in affective disorders, may predispose towards lowered serotonin synthesis in the brain (Maes et al., 1991b, 1996, 1997a). All in all, the lowered TRP concentrations are at least in part the consequence of IRS activation in MDD/BD and, in fact, constitute a CIRS response aimed to attenuate hyperinflammation and combat infections (Maes et al., 2011d). Other mechanisms for decreased TRP availability include a) platelets being activated in MDD (Moreno et al., 2013), which may be accompanied by an increased TRP uptake; and b) the circulatory levels of free fatty acids, which are in part mediated by insulin levels (Almulla et al., 2022c). Because serum/plasma TRP availability influences TRP concentrations in the brain (Curzon, 1979; Fernstrom et al., 1973), decreased TRP concentrations in peripheral blood may influence serotonin synthesis in the brain, which is thought to play a role in MDD/BD (Coppen, 1967; Lapin and Oxenkrug, 1969; Maes and Meltzer, 1995; Oxenkrug, 2013).

The lowered levels of TRP may suggest that MDD/BD patients show lowered neuroprotection. First, TRP and serotonin are antioxidants (Xu et al., 2018), and serotonin has neuroprotective properties by preserving neuroplasticity and preventing neuronal injuries (Croonenberghs et al., 2005; Rădulescu et al., 2021). In fact, the antioxidant role of TRP is indirect (Pérez-González et al., 2015; Perez-Gonzalez et al., 2014) via metabolites such as melatonin, serotonin, 3-HK and XA (Reiter et al., 1999). Second, low levels of some TRYCATs, including KYN, KA, XA and QA, may negatively impact neuroprotection because these TRYCATs have anti-inflammatory effects for example by lowering the IFN-γ/IL-10 ratio (Maes et al., 2007), whilst KA, 3HK, 3HA, and XA have antioxidant effects (Goda et al., 1999; Maes et al., 2011d). In addition, KA has a neuroprotective role by downregulating the excitatory receptors in the brain, namely, N-methyl D-aspartate (NMDA), α-amino-3-hydroxy-5-methyl-4-isoxazolepropionic acid (AMPA), and kainate glutamate ionotropic receptors and by impeding the alpha 7 nicotinic acetylcholine receptor (Morris et al., 2016).

### 4.5 Limitations

This study has shown a number of limitations that should be considered in future investigations. First, few studies reported on the central levels of TRP and TRYCATs and, therefore, more research should focus on postmortem tissue and CSF concentrations of TRP and TRYCATs. Second, a substantial proportion of the studies included in our analysis lacked information about the treatment histories of patients. This may be relevant because at least some antidepressants, such as escitalopram, are associated with a lower plasma KYN/TRP ratio due to a decrease in KYN but not TRP (Sun et al., 2020). Thus, it is preferable to thoroughly investigate the TRP and TRYCATs in drug-naïve patients with a first episode or to control statistically for the medication status. Moreover, future research should stratify patients according to the staging phases of affective disorders since disease staging is related to greater neurotoxicity (Maes et al., 2019b, 2022a). Fourth, a considerable number of studies seemed to have been conducted without correcting for major confounding variables such as smoking and alcoholism and their effects on TRP (Badawy, 2002) and TRYCAT (Leclercq et al., 2021) metabolism. Importantly, it may be that IDO activation is a hallmark of somatization rather than of affective disorders (Maes et al., 2011c) and, therefore, future research in affective disorders should always include measurement of somatization and examine the comorbidity between MDD/BD and somatization. It may be that the assay of immunoglobulin A (IgA) responses to TRYCAT adducts is much more sensitive than the methods used in the studies included in our meta-analysis. Thus, while the neurotoxic TRYCATs assessed with the conventional methods were not associated with schizophrenia (Almulla et al., 2022c), we found that IgA responses to neurotoxic TRYCATs were strongly associated with the severity of the phenome of schizophrenia (Kanchanatawan et al., 2018).

The majority, if not all, studies compared TRP and TRYCATs between patients diagnosed with a major depressive episode per DSM or ICD criteria and controls. Recently, however, we have demonstrated that such diagnostic criteria are grossly inadequate (Maes and Stoyanov, 2022) and that precision nomothetic psychiatry has enabled the construction of an endophenotype class of severely depressed patients (termed Major Dysmood Disorder) with immune-inflammatory and nitro-oxidative disorders (Maes et al., 2019a, 2022b; Maes and Stoyanov, 2022). This is the target class for detecting IDO-induced changes in TRYCATs.

## 5 Conclusion

The key findings of the present systematic review and meta-analysis are summarized in Fig. 1. Both MDD and BD are probably associated with central and peripheral TRP depletion, which may be explained by lowered serum albumin levels. IDO enzyme activity did not exhibit signs of hyperactivity, as indicated by the patients' lowered KYN levels and an unchanged KYN/TRP ratio. Moreover, there is no evidence that MDD and BD are accompanied by increased neurotoxicity due to an activated TRYCAT pathway. Future research should employ the precision nomothetic approach and control for the use of antidepressants (Maes and Stoyanov, 2022) to delineate the involvement of serum and CNS (not plasma) TRP and TRYCATs in Major Dysmood Disorder and not a major depressive episode according to DSM/ICD criteria. Moreover, future studies should a) focus on the probable causes of lowered TRP availability to the brain and unchanged IDO activity despite immune activation in mood disorders (including effects of nitrosative stress), and b) re-examine IDO activity and TRYCATs in association with staging of illness, which is largely mediated by immune and oxidative stress pathways (Maes et al., 2019b, 2022a).

## Ethical approval and consent to participate

Not applicable.

## Consent for publication

Not applicable.

## Availability of data and materials

The last author (MM) will respond to any reasonable request for the dataset (Excel file) employed in the current meta-analysis after it have been fully exploited by all authors.

## Funding

The study was funded by the C2F program, 10.13039/501100002873Chulalongkorn University, Thailand, No. 64.310/169/2564.

## Author's contributions

The study was designed by AA and MM. AA, YT, and AV collected the data. AA and MM performed the statistical analysis. All authors contribute to the writing of the paper and approved submission of the final draft.

## Declaration of competing interest

The authors declare that they have no known competing financial interests or personal relationships that could have appeared to influence the work reported in this paper.
